# Platform workers and digital agency: Making out on three types of labor platforms

**DOI:** 10.3389/fsoc.2023.1063613

**Published:** 2023-03-27

**Authors:** Tuomo Alasoini, Jere Immonen, Laura Seppänen, Marja Känsälä

**Affiliations:** Finnish Institute of Occupational Health, Helsinki, Finland

**Keywords:** agency, algorithmic management, autonomy, control, labor platform, making out, platform work

## Abstract

Much of the research on platform workers has focused on individuals involved in low-skilled and highly standardized tasks. However, platform workers are not a homogeneous group. Utilizing a classification system that makes a distinction between different layers of platform control and grouping platforms according to how they divide decision rights between platforms and workers, we examine how and for what purposes platform workers operating in three types of control contexts have practiced and developed their digital agency for making out. The study, based on an analysis of platform webpages and 32 semi-structured interviews of food couriers, freelancers, and interim managers, shows that workers can exercise their digital agency on all three types of platforms, but different platforms create different conditions for this depending on their special forms of control. In addition, the forms of control also affect to what extent workers are motivated to direct their agency for making out. Instead of regarding platform work as just another layer of a periphery segment in the labor market, our analysis suggests that platforms exercising algorithmic control are new types of arenas for work, which seem to reproduce, or even amplify, the inequalities found in the offline world of work in the digital world.

## 1. Introduction

Autonomy and control have been key concepts in sociological studies analyzing the labor process. In labor process theory, a theoretical framework inspired by Braverman's (1974) seminal work, employers' need for control and workers' struggle for autonomy have been examined through Marx's well-known distinction between labor and labor power. Autonomy and control have also been important topics in mainstream sociology of work. In his classic ethnographic study, Roy (1954) depicts how manufacturing workers strive to maintain a counter-system of control for “making out” in response to managers' efforts to narrow their shopfloor autonomy. The issues of autonomy and control have played an important role in experiments inspired by sociotechnical systems theory as well. In fact, the idea of a (semi-)autonomous group has been the most common (re)design feature of sociotechnical projects promoting quality of work and industrial democracy (Guest et al., 2022).

Computerization has changed the context of sociological debate on autonomy and control in work, and the Internet has become the basis of a globally available information space, and a new sphere of social action and for interconnecting people. Much of the research has analyzed such development as a digital revolution of work, which—besides requiring new skills—enables new forms of managerial surveillance (e.g., Briken et al., 2017; Zuboff, 2019; Moore and Woodcock, 2021). Like workers in all previous stages of industrial history, also workers in the digital environments can develop strategies of “making out” for securing higher earnings, clearing up space for autonomy in work, or fiddling the management system for their own benefit. However, workers' acts of “making do” vs. “making out,” i.e., workers' exercise of their labor power *with* vs. *against* the grain of corporate intentions, are often intertwined and difficult to disentangle, containing elements of both consent and resistance (McCabe, 2014).

Platform workers are an example of a new group of workers whose work is largely managed *via* smart technologies, such as algorithmic allocation of tasks, monitoring and tracking devices, and ranking and rating tools. The focus of sociological research has been on new forms of algorithmic control and their putative degrading effects on work, information asymmetries between workers and platforms, and the lack of safety nets in typical platform work without the benefits and protection provided by standard employment relationship (e.g., Bergvall-Kåreborn and Howcroft, 2014; Stewart and Stanford, 2017; Lehdonvirta, 2018). At the same time, less attention has been paid to the new skills and coping strategies that workers can develop as their knowledge of the operation and logic of the control systems grows. Working with such systems can help workers also to cope with other kinds of digital work environments for the future.

This paper combines conventional sociological research interest in autonomy and control in work with research interests deriving from educational studies on digital agency (Passey et al., 2018) and communications studies on digital inequality (Helsper, 2012; Van Deursen et al., 2017). We explore in Finnish context the ways in which workers on three types of labor platforms seek to maintain and increase their autonomy and opportunities for making out. The paper contributes to existing research in three main ways. First, it uses “digital agency”—a concept developed in educational sciences but repurposed for our sociological inquiry—as a framework for analyzing and contextualizing platform workers' coping activity. In this way, the paper aims to increase the conceptual understanding of the workers' opportunities to practice agency in platform work. Second, the paper contributes to existing research on the autonomy/control nexus in platform work by analyzing the workers' agency in a context where control is viewed as a multi-layered phenomenon. By doing so, it aims to shed light on the role of the platforms' control structures for factors enabling vs. constraining the workers' conditions for practicing agency. Finally, the paper brings similarities and differences in the opportunities to practice agency between workers operating *via* three types of platforms into a systematic comparison with each other, thereby illustrating the reproductive nature of the platforms for social inequalities in the labor market. Choosing three different types of platforms for the analysis enables a versatile parallel examination of the wide spectrum of platform control structures.

Although the share of platform workers among all employed people in developed industrial countries is still relatively small, we consider it important to better understand the logic of labor platforms and its implications for the workers' autonomy, agency, and coping strategies. Platform companies are forerunners in algorithmic management, but it is expected that its applications will increasingly spread to more traditional forms of work, having a major impact across the economy (Fourcade and Healy, 2017; Zuboff, 2019; Gilbert and Thomas, 2021). The COVID-19 crisis also gave an extra boost to platform companies, many of which profited from the crisis and conquered markets from more traditional players (Schaupp, 2022). With the help of our paper, we want to draw attention to the multifaceted nature of platform work and its control structures, as well as their importance for the future quality of working life, social inequalities in the digital labor market, the needs for and possibilities of the regulation of platform work, and new potential areas for academic studies.

The article is organized as follows. The next section elaborates the research questions and research tasks and the underlying theoretical discussions. This is followed by a description of the data and methods. Thereafter, the results are presented. Finally, the results, contributions, and limitations of the study are discussed.

## 2. Theoretical foundations, research question and research tasks

Scholars have developed different classifications of labor platforms as well as different images to describe and analyze platform work (Vallas and Schor, 2020). However, much of the research on platform workers has focused on individuals involved in low-skilled tasks, such as food couriers, ride-hail drivers, or micro-taskers, as showcases of precarious “digital labor.” At the same time, those doing more demanding knowledge work *via* online freelancing platforms have been the subject of less sociological studies (however, see Schörpf et al., 2017; Nemkova et al., 2019; Sutherland et al., 2020; Rahman, 2021; Seppänen et al., 2021; Wood and Lehdonvirta, 2022). A recent study in Denmark found out that, of all platform workers in the country, about one third were “established workers” many of whom were able to combine platform work with high-skilled and well-paid jobs in the conventional labor market (Kristiansen et al., 2022).

Given the fact that platform work is a multifaceted phenomenon, also the autonomy/control nexus can take different forms in such work. In the following, we would like to emphasize the importance of three aspects that are particularly relevant from the perspective of our paper.

### Separation of power and control

First, in platform work that is performed outside standard employment relationship, the context of the autonomy/control nexus differs from that of typical work performed in such a relationship. Labor platforms' modes of control and organization differ in this respect, not only from traditional organizational hierarchies, but also from markets and (collaborative) networks (Aneesh, 2009; Kellogg et al., 2020). Platforms delegate and distribute control among the participants—including clients and workers—but, at the same time, they centralize power (Vallas and Schor, 2020). In other words, a discussion of autonomy and control in platform work reaches only to a limited extent the elements of power associated with such work. From the perspective of our paper, this means that by analyzing different platform control structures, we cannot draw straightforward conclusions regarding the distribution of power between the different parties on different types of platforms.

### The dual nature of control

The observation of control as delegated and distributed leads to our second aspect. Platform workers face dual control that comes from the direction of the platform on the one hand and the direction of the client on the other. The specific forms of control and their weighting between these two sources may greatly vary between platforms. This duality, which finds its mathematical embodiment in the platform's algorithmic management, can be a major concern for the workers' reputation and access to new tasks, depending on the specific characteristics of the platform's control system (e.g., Schörpf et al., 2017; Rahman, 2021; Wood and Lehdonvirta, 2022). Another duality of control in platform work is its simultaneous technological and normative basis. Gandini (2019) talks about techno-normative control that platforms set upon workers. Techno-normative control is articulated in two main ways: on the one hand, client-led practices that rank and rate workers, and, on the other, digital monitoring and surveillance of work performances by the platforms. These two manifestations of control also limit spaces where workers can practice and develop their agency.

### Control as layered

The final aspect relevant to our paper concerns platform control as a layered process. Here, our main source of inspiration is Maffie's (2020) division, which distinguishes between three stages of control. The first stage (architecture of identity) covers how a platform creates a traceable identity for each platform user. In the second stage, the platform centralizes the key elements of an exchange, such as trust, communication, and payment. Finally, the platform can exercise market control and set the terms under which workers and clients enter in an exchange. Platform centralization builds on the architecture of identity and market control, in turn, on centralization. In practice, labor platforms differ greatly in how far they go in this process.

We utilize the concept “digital agency” to map out special features that workers need for making out on platforms. Digital agency refers to the individual's ability to control and adapt to a digital world, comprising three elements (Passey et al., 2018). Besides digital competence, digital agency requires digital confidence (in brief, utilizing one's digital competence in different digital domains in an agentic way) and digital accountability (in brief, utilizing one's digital competence responsibly and ethically). Digital agency can be considered transformative when workers, either individually or collectively, have a capacity to voluntarily form and implement intentions that go beyond and transform the accepted routines and given conditions of activity (Vänninen et al., 2015). Transformative agency helps workers to maintain a sense of meaningfulness in their work and activities. Besides tangible actions, such as making do/out, it includes reflection, sensemaking, and planning.

Platform workers' digital agency does not have to question the power exercised by platforms. However, digital agency gives workers the *potential* to deviate from, or act against, the grain of platform intentions that they perceive unfair and look for and learn about loopholes or vulnerabilities involved in the control system to pursue their own goals. Examples of this kind of activity can be found in several studies on platform workers (e.g., Shapiro, 2017; Galière, 2020; Kellogg et al., 2020; Bronowicka and Ivanova, 2021; Heiland and Schaupp, 2021; Seppänen et al., 2023). We consider this activity through which workers can gain extra space and freedom in their work here as the core manifestation of workers' ability to make out. In that sense, making out can be considered not so much as resistance *to* the (control) system, but rather resistance *within* the system (e.g., Roy, 1954; Burawoy, 1979; McCabe, 2014; Perrig, 2021).

The research question is derived from the above theoretical discussions. The research question is how and for what purposes platform workers operating in three different types of control contexts have practiced and developed their digital agency for making out. We consider workers' agency as a particularly relevant research topic in the analyses of those working on all types of labor platforms for two reasons: first, like other independent workers, platform workers must compensate for their lack of organizational support with their own complementary personal arrangements, and second—what is special for platform workers also compared to other independent workers—platform workers operate in a new ecosystem that includes many uncertainties and opacities (Sutherland et al., 2020). Moreover, we consider that the issue of *digital* agency is of crucial importance for platform workers, even if their actual work contains physical performances, because many key stages of the task allocation/acquisition and the actual work process are technologically mediated and algorithmically controlled (Curchod et al., 2020; Kellogg et al., 2020).

To answer the research question, we set ourselves two research tasks.

*First*, we examine what forms of control there are on different labor platforms and how and to what extent the platforms differ from each other. The analysis utilizes Maffie's (2020) classification. Its advantages, from the perspective of our paper, are that it is one of the few classifications that makes a distinction between different layers of platform control and explicitly groups platforms based on how they divide decisions rights between platforms and workers. Based on the classification, we distinguish six dimensions of control of which performance evaluation, centralization of worker-client communication, and centralization of payment demonstrate how platforms centralize exchange. Filtering, price setting, and matching, in turn, are analyzed as aspects of market control exercised by platforms. We assume that workers can exercise their digital agency on all types of platforms, but different platforms create different conditions for this depending on their special forms of control (Sutherland et al., 2020; Veen et al., 2020). Our assumption is that the more work performance can be standardized, the less room it leaves for worker agency and, in this way, constrains workers' chances of making out. This assumption is also in line with the stratification hypothesis presented in communications research. The hypothesis suggests that the digital world of work reproduces existing social inequalities by replicating offline structures and because offline human capital carries over to the digital world (Van Deursen et al., 2017).

*Second*, we examine how workers have practiced their digital agency in different platform control contexts. Different contexts may give rise to different manifestations of digital agency depending on, among others, how fair workers consider them to be. The fairness of platform work practices is an important and rising research theme (e.g., Faullant et al., 2017; Graham and Woodcock, 2018; Fieseler et al., 2019; Pfeiffer and Kawalec, 2020; Shanahan and Smith, 2021; Seppänen et al., 2023), but here we only touch upon it as part of answering to our research question. We assume that the less fair workers perceive platform operations, the greater motivation they have to practice and develop their digital agency for making out.

## 3. Materials and methods

We selected three types of platforms for this study on the grounds that they mediate different types of work and assuming that the nature of the mediated work is associated with the forms of platform control and through this to workers' use of their digital agency. The selection was preceded by an analysis of webpages and terms of service of 46 labor platforms operating in Finland, where they were examined whether their human resource management follows more market vs. corporation logics (Immonen, 2023). In that study, the platforms were divided into six models based on the emergence and characteristics of those logics and the variations between them. The grouping provided us with foreknowledge of platform forms of control. The type descriptions in Subsection 4.1 were formed based on a combination of an analysis of webpages and interview data using Maffie's (2020) terminology.

As the first type, we selected food-delivery platforms that convey standardized work requiring low skills, represented in our material by Alpha and Beta (pseudonyms). The second type in the study is a global marketplace Gamma (a pseudonym). The tasks mediated through Gamma can be characterized as skilled knowledge work performed by professionals. The third type is a smaller Finnish platform Delta (a pseudonym) specialized in interim management.

The main sources of information in this study were semi-structured interviews with platform workers and an analysis of the platform webpages. Interview participants were identified in two main ways in connection with two research projects. Freelancers working *via* Gamma were selected among respondents to an online survey who indicated that they were also willing to be interviewed. Food couriers and experts working *via* Delta were acquired in collaboration with the companies. Invitation letters prepared by the researchers were sent through the companies' information systems, and workers contacted the researchers directly using the contact information provided in the invitation. At every stage, it was underlined to the workers that the companies were not involved in the research and did not know who had signed up for the interviews. The first registrants were selected to be interviewed. The total number of interviewees was 32. The interviews were conducted at different times depending on the platform.

All 10 food couriers were interviewed in 2021 and 2022 *via* Teams or phone. The length of the interviews varied from 50 to 90 min. Nine of the interviewees were men, and eight of them had an immigrant background. Eight of the couriers handled deliveries by car, one by bike, and one by both, depending on the situation. Half of the couriers had experience working *via* both platforms. At the time of the interview, three worked only for Alpha and four only for Beta. The interviewees differed greatly in status and platform activity. Five of them also had full-time or part-time employment elsewhere, three were students, and only two were full-time self-employed. Four worked through the platform daily, four weekly such as weekends, and 2 monthly or less frequently.

The Gamma material included 15 interviews. The interviews were conducted in 2018 and 2019, lasting 40–80 min. One of the interviews was conducted face-to-face and the others *via* Skype. Most interviewees were men (*N* = 11) and native Finnish speakers (*N* = 10). Here, also, the interviewees formed a heterogeneous group in knowledge area, status, and platform activity. The biggest occupational categories were translators (*N* = 4), graphic designers (*N* = 3), and consultants (*N* = 3). Seven of the professionals were full-time self-employed, whereas five were in salaried employment elsewhere, two were students, and one was unemployed. Three worked through the platform daily, five weekly, four monthly, and three less frequently.

Seven people who had experience working through Delta were interviewed in 2021 and 2022 *via* Teams. The interviews lasted 45–90 min. All interviewed were native Finns, five of them men. All the interviewees had an academic degree and long-term experience working in various business management tasks, also as salaried employees. They all had own business name, but it was not uncommon that their recent careers included alternating periods as interim and salaried managers or other high-level specialists. At the time of the interview, two were in employment relationship to a company. Their typical assignments were long, spanning months.

One of the authors conducted all interviews of persons working through Gamma and for whom Alpha was the main platform, and another author all other interviews. The interview protocol was initially modified from a study by the Institute for the Future (Anderson and Westberg, 2016). It started with mapping of the workers' basic background information and the significance of platform work for them. Thereafter, the interview form was divided into themes, including the platform operations and workers tasks, transactions, interaction/communication, control of and support in work, problems, freedom of action, learning opportunities, fairness, and the role of society. Each main theme was further divided into subthemes and guiding questions. The questions were in each case modified to better address company-specific factors. The interviews were recorded and transcribed with the permission of the interviewees, and the transcripts were used in the coding process.

The interview data analysis proceeded as follows:

First, the interview data were roughly coded using the ATLAS.ti software by three of the authors into three categories of agency by naming them “expressions of tangible actions,” “reactions to problematic issues,” and “the future.” The third category was inspired by Emirbayer and Mische's (1998) article on agency. In all three categories, the focus was on agency that expresses the relation between the worker and the platform. The analysis method can be called abductive (Paavola, 2021), meaning that both empirical insights from the data and theories of agency (see above) were used in the analysis. Each interview was first coded by one of the authors and thereafter discussed together. After several rounds, one author checked all final encodings.

Thereafter, citations/episodes demonstrating the three categories were recoded into “questioning,” “making do/out,” and “ideating.” “Questioning” included criticism of, resistance to, reflecting on, or questioning actual problematic issues. Questioning can be considered a trigger for taking other agentic actions. Making do and making out are action-oriented types of agency, where the interviewees tell about something they do, have done, or are planning to do in the future. However, the episodes in this category differ from mere declaratory speech through the deep feelings, commitment, or desire involved that indicate how the content is relevant or important for the interviewees. We first looked at acts of making do/out as one category before making a more fine-grained distinction between them in the analysis. “Ideating” looked at the interviewees' future developmental intentions in their work contexts. It was conceptualized as suggested improvements to other people, to the platform, or to other organizations or institutions. Together, the three categories of questioning, acting (making do/out), and ideating form a model of potential steps of changing or transforming the normal course of work activities (Heikkilä and Seppänen, 2014; Vänninen et al., 2015). However, because of the diversity of the topics involved in agentic episodes around the worker-platform relation, we do not know whether, to what extent, or how the issues of questioning turn into actions or ideating. The categorization only shows us the qualities of agency in the data.

Finally, the analysis was concluded in two parts: as an overview of the three categories of digital agency and a more detailed examination of acts of making do/out in the three types. In all categories, the prefix “digital” was considered in a broad way and all agency taking place in the platform environment was included. This is justified, as algorithmic tools and systems significantly mediate and shape workers' possibilities for practicing agency on the platforms.

## 4. Results

In the following, we present our results in three subsections. We first present the division of platforms into three types based on Maffie's (2020) classification. Next, we examine what forms of worker agency were found in the interview text material. Finally, we give examples of episodes of making out in the data.

### 4.1. Autonomy and control on platform labor exchanges

The results of the first research task, which focused on the forms of control on different labor platforms, are summarized in Table 1. As was our initial assumption, the platforms clearly differ from each other in many of the dimensions of Maffie's (2020) classification, forming three distinctive types; the two food-delivery platforms operate on largely similar principles. In the following, the types are described in more detail.

**Table 1:** Forms of control on three types of labor platforms.

	**Type 1**	**Type 2**	**Type 3**
Performance evaluation	Client ratings (Alpha and Beta) and a ranking system based on them (Alpha)	Client ratings and a visible Score based on them	No
Centralization of worker-client communication	Yes: only limited worker-client communication	Yes: control of worker-client messaging	Yes: control of worker screening and worker-client negotiations
Centralization of payment	Yes	Yes	Yes
Filtering to clients	No	Yes	No
Price setting	Platform-determined	Negotiated between worker and client	Negotiated between worker and client
Matching	Forced	Open	Single-sided

*Type 1*. The two food-delivery platforms represent the first type. Both control the number of workers registered on the platforms, but neither has explicit eligibility criteria for becoming a courier. Exchange on the platforms takes place as an interaction between clients, couriers, shops or restaurants, and the platform. Platforms' control structures also affect the autonomy of shops and restaurants in many ways, but, here, the focus is exclusively on couriers.

Both platforms have performance evaluation systems for workers based on client reviews and data collection on work performances. In Alpha, both client ratings and the data collected by the platform affect the couriers' inner rankings within the platform, affecting their possibilities to get new work shifts. Couriers are ranked every 2 weeks in a way that determines the order in which they can book shifts for the forthcoming days, i.e., those ranked highest are first in line. Beta used to have a similar procedure, but it changed the system so that couriers can now sign in to work whenever they want. Common to both platforms is that it is not completely clear for workers how client reviews and the collected data are used in the platforms' algorithmic management procedures and task allocation. Beta's policy is more transparent, and it has informed couriers that their availability for performing the task and their distance from the pick-up place are the only influencing factors in task allocation. Beta also collects data on work performances but for monitoring potential neglects related to deliveries and not for ranking couriers.

Communication on both platforms is centralized, leaving in normal cases only little need for interaction between clients and couriers. Clients and couriers can communicate by phone only in the final stages of the delivery when food or product is brought to clients. In other stages, communication is supposed to take place *via* the platforms' help desks. Both platforms use forced matching where they exclusively set the rules of work allocation and allocate tasks to potential workers by using algorithmic management. Couriers operate anonymously, and clients cannot influence who handles the order.

Both platforms also exclusively determine consumer prices and the level of compensation for couriers of made deliveries. In both cases, it is up to the platforms to decide through algorithmic management how many and what kind of tasks workers receive, directly affecting workers' earnings. Both platforms use economic nudges to match supply with demand, in addition to which Alpha directly controls supply through its shift booking system. Beta pays all couriers only for made deliveries, whereas Alpha applies two types of contracts. Some couriers have old contracts that guarantee them a basic salary for made hours added with payment for made deliveries, whereas recently joined couriers are paid only based on the number of deliveries.

The overwhelming platform control over matching and price setting means that both platforms do not act only as market creators, but they also have an undisputed role as market regulators (Maffie, 2020).

*Type 2*. The second type is represented by Gamma, a global marketplace where clients can search and hire skilled freelancers from various fields of expertise, like design, marketing, engineering, coding, or translation. All projects are performed online. Skilled freelancers all over the world can compete for the same projects unless the client wishes to restrict this for some reason. The platform also regulates the supply by limiting the number of registered freelancers in the same field of expertise. Gamma does not control the progress of actual work performance in as straightforward way as food-delivery platforms or involve in work intermediation and negotiations between clients and freelancers.

The cornerstone of Gamma's control system is its rating procedure that enables clients and workers to evaluate each other after the project ended. Clients' five-scale ratings form the basis to a worker's score, which is visible to all clients and on which her/his online reputation is based. Those with highest ratings and the thereby achieved badge showing success have an advantage on the platform when competing for new assignments. Besides the score, filtering is affected by other things, such as freelancer's activity on the platform, the number of projects completed, and possible competence certificates. Gamma offers workers different options to attach to the platform. In normal cases, Gamma limits the number of projects to which workers can apply in a month but by paying an extra fee workers can raise the number.

While matching occurs quite freely without the platform intervening, communication between clients and workers is closely supervised by Gamma. Messaging between clients and workers is monitored by filtering expressions that may involve contact information. The reason for strictly controlling transactions between the parties is that payments must be managed through the platform and the service fee taken by the platform is tied to a freelancer's earnings from a client. Gamma does not control work performances as such, but in hourly-paid projects it allows clients to supervise working through an app that takes screenshots of the worker's desktop every few minutes. Such a technological monitoring is intended both to enable clients to supervise working and to certify workers' right to the agreed fee. Gamma does not directly restrict workers' autonomy by participating in job design or determining workers' price requests or levels of compensation. Negotiation on terms of the projects is left to clients and workers.

*Type 3*. Delta represents the third type. Unlike the previous types, the platform does not control actual working in any way but has an important role in work allocation. Delta does not have formal eligibility criteria, and basically anyone can register as an expert suitable for demanding management tasks. However, before experts are allowed to present their profiles with named references and apply for projects on the platform, Delta checks the authenticity of the information. The projects are client-determined and often require high-level expertise and extensive experience from workers. Thus, workers often have great autonomy in their projects, but—unlike freelancers in Gamma—the nature of the tasks also require occasional onsite working.

The platform's role is emphasized in work allocation, which distinguishes this type from the two others. Matching is neither forced nor open, but it can be characterized as single-sided. In this type of matching, all registered experts can apply for a project, but the platform screens the applicants and decides which of them will be presented to the client anonymized. In this way, the platform maintains extensive power over who will have access to a project. The purpose of anonymization is to prevent discrimination and to contribute to the fact that the selection takes place purely based on the merits of the applicants. During matching, communication between worker and client is centralized, as in the other types, meaning that negotiations between clients and workers are also managed through the platform. Terms of working and compensation are also negotiated on a tripartite basis between clients, workers, and the platform.

While work performances are not controlled by the platform, payment transactions must be handled through the platform's payment system. However, clients and workers can also sign contracts in which payments take place pass the platform, but, in this case, clients must pay a special exit fee to the platform. Compared to the previous types, in type 3 the platform plays a lesser role in the actual work process. The platform's controlling role is emphasized in matching, which requires a lot of traditional manual work from the platform staff. Single-sided matching for this type of demanding expert projects still requires human judgement and cannot be left to algorithmic decision making.

### 4.2. Digital agency on three types of platforms

This subsection examines expressions of workers' digital agency in the three types. Applying the categorization presented in Section 3 to the analysis of transcribed interview text data, 488 episodes in total were found, showing a clear difference in their distribution between the types (Table 2). Questioning was prevalent among couriers, whereas making do/out was the most common type of expression in the two other types.

**Table 2:** The number of episodes of digital agency in three types of labor platforms.

	**Type 1**	**Type 2**	**Type 3**
Questioning	111 (49%)	85 (42%)	14 (24%)
Making do/out	69 (31%)	95 (47%)	31 (53%)
Ideating	48 (19%)	22 (11%)	13 (22%)
In total	228 (100%)	202 (100%)	58 (100%)

*Type 1*. In the food-delivery data, nearly half of the episodes contained questioning, most targeting the platform. The episodes dealt with multiple issues, but, here, we briefly describe only two of them. The first concerned platform practices, focusing on the ways couriers were controlled, insufficient communication between couriers and the platforms, unexpected changes in the terms of couriers' contracts, the platforms' abstinence to encourage interaction between couriers, and inflexibility in cases of problems with weather. A discrepancy between the platforms' marketed freedom and couriers' status as independent workers, and their experiences of being controlled like employees was high on the list. The second major issue concerned competition between couriers. In Alpha, couriers are ranked according to their work performance into levels that determine their chances to choose work shifts. However, many factors affecting their performance are beyond their reach. A courier may have a long relationship with the platform, but, still, only the latest 2-week period determines her/his ranking, a fact considered unfair by many. In Beta, couriers are free to work whenever they want. This caused complaints about long waiting times and wondering about Beta's continuous recruiting of new couriers. Couriers on both platforms expressed that they do not know how tasks are allocated to them. As agentic actions to questioning issues, couriers tried to find out how matching operates by analyzing their gigs, asking questions from the platforms' support centers, or discussing with their colleagues.

Acts characteristic of “making do” were found in the ways workers combine courier work with their family life, studies, or hobbies, how they use platform techniques for their benefit, and their attempts to work faster. Working faster enables them to take more assignments and earn more money, and, in Alpha, every additional assignment increases their chance of attaining a higher ranking. Here, couriers act according to platform intentions, but they still experience autonomy in doing so. Acts of “making out,” in turn, are seen in couriers' tricks or shortcuts in bending the rules of the platforms or combining working with both platforms for their own benefit (see Subsection 4.3).

The focus of “ideating” was on platform practices and technologies, including in some cases also considerations of benefits for platform companies and clients. The couriers wished for more information and better visibility about algorithms in rankings, payments, and task allocations. Higher payments were also wished, but many couriers regarded that their level of earnings very much depends on themselves. Restriction to the number of couriers, greater respect for them, and the possibility to have a steady salary were typical issues on couriers' wishing list to the platforms.

*Type 2*. Questioning issues that directly affect income from the platform—client ratings, obscure ranking system, and (global) competition over assignments—was frequent among freelancers. A major source of dissatisfaction was the high service fee taken by Gamma. As a counter strategy, freelancers developed their competence levels, sought for clients also from other sources, considered leaving the Gamma platform, or collaborated with clients outside the platform.

While couriers' interaction with clients is thin and short-lived, platform practices in Gamma encourage freelancers to develop long-term relations with clients in different ways. Despite the fact that freelancers saw platform services in a relatively positive light, these also raised criticism among them. It was widely believed that platform services favor clients at the expense of freelancers, and the reciprocal rating system has the potential to sanction the reputation of freelancers much more than that of clients. Such experiences of injustice gave rise to multiple forms of gaming behavior, as will be described in the next Subsection.

“Ideating” in the case of freelancers largely centered on how to diminish competition from low-cost countries and with lower-skilled freelancers on assignments, such as founding new platforms operating only in Finland. Freelancers also hoped for better possibilities for networking with their online peers and more information about clients and platform practices and rules.

*Type 3*. Experts working *via* Delta also questioned the imbalance between supply and demand on the platform. There is only a limited number of assignments available for hundreds of people looking for them. The platform's practice of anonymizing applicants was also questioned as well as the platform's rather undeveloped level of technology. Unlike Gamma, which is global and maintains arm's length relationships with freelancers, Delta operates locally and organizes social events where experts and clients can meet each other. Delta's encouragement of experts' social networking was applauded, but its business impact on the platform was also questioned.

Building social relations with clients and occasional rotation between platform work and employment with the same clients are typical examples of “making do” by experts. The dense social networks prevailing between the platform and many of the experts and clients raise the threshold for gaming behavior among experts, radically distinguishing type 3 from the previous two. In some cases, this may even have led to mixing of roles between the parties, such that experts have acted as advertisers of platform services to potential clients. However, manifestations of making out can also be found. At least in one case an expert had negotiated directly with a client about getting a project. This is against Delta's protocol, but it was apparently possible with its tacit agreement.

The experts' development ideas did not primarily relate to how the position of those working *via* platforms could be enhanced, but rather how platforms could improve their (customer) service. This is understandable given their experience working in management positions. Many of the ideas also focused on the gig economy more broadly and not so much Delta.

### 4.3. Episodes of making out: Examples

Here, we present typical episodes of making out found in our data. The examples are under four headings with some example quotes gathered in Table 3 (see references to them in the text). As we know from previous literature (McCabe, 2014), and as the examples below will show, drawing a line between making do and making out is not straightforward.

**Table 3:** Example quotes of four types of making out.

	**Example quotes**
Working on multiple platforms	Quote 1: “If everything is okay with [Beta], then I go for Beta, but if there is not [work], then I go for Alpha. The problem with Beta is there is no limit, everyone can start working any time. So, it happens like tomorrow if I go at 12 o'clock, the lunch time, I won't get much task, because there are many people working for Beta. But with Alpha you have to book a shift, then they already have predicted how many tasks will come this weekend” (Alpha/Beta).
	Quote 2: “If a project matching to your profile becomes available, you should apply if you have the time and resources. But then again, because projects appear so infrequently and there is a large number of members on the platform, it is worthless to try even if you have time, because you will not get another from there if you have one active project running. But, of course, you can try from the outside, which is why I am also in that other service” (Delta).
Taking on new roles	Quote 3: “Well, it [registration also as a client] definitely gives you a sense of your competitors and what's out there. Especially if you use it from both sides as a client and a freelancer. I think it's really the key, you need to know how it works from both sides. If you don't do that, you're missing out of 50 per cent on the platform and how it works. So, using it as a client helps me understand my competitors… […] And that's a really huge asset for anyone who is on [Gamma], understand what goes on there on the other side” (Gamma).
Tricks	Quote 4: “The relation to these companies is so distant as a courier that you do it for yourself. I don't take any responsibility to promote the company because it's not that fair. If I was a paid employee and I had professional pride and security and facilities where to see colleagues, that kind of work community, so then it would be a totally different matter” (Alpha/Beta).
	Quote 5: “You can trick so that there comes a gig that you don't want to take, so you can ask for a break in the app, for example a 5-min break. And if it's not busy, they accept it without… And then that order goes away. And you are on a break. And when that gig has gone away, you stop that break immediately, and then you're working again, and there comes new gigs again” (Alpha).
	Quote 6: “I asked a friend to hire me on the website, so I could have a job listed on there, and he had actual work, he needed me to look at his résumé. I said, “look, I'm gonna lose 20 percent but could you please at least hire me through Gamma so I would have the job rating,” and he said “sure.” We went through the process, and then afterward, he told me how it works, explained behind the scenes what he was seeing and how many people were applying for the job” (Gamma).
Disintermediation	Quote 7: “But these fixed-priced projects are entirely different things. I don't often advise clients that we shouldn't take them through [Gamma], but I do offer them a discount if we don't. Because then we will both save money on labor costs and it's often fine for them” (Gamma).

#### 4.3.1. Working on multiple platforms

Many food couriers had experience working on both platforms under study. They can switch platforms by assessing which one best serves their interests at each point of time, as illustrated by quote 1 in Table 3. As one platform offers potentially more gigs on busy times and the other predictability, the worker strategically moves between the platforms depending on demand. This strategy involves questioning each platform's downsides. The objective of this kind of making out by using two platforms is to avoid waiting times and maximize income and work autonomy.

Most interviewed experts on Delta also utilized multiple channels for marketing their services and expertise, including other platforms. The motivation for such a strategy of decentralization was both the small number of projects available *via* the platform and questioning of the platform's assumed operational logic. The assumption was that the platform strives to allocate projects to experts evenly, putting especially those with running projects at a disadvantage when competing for new projects. Using multiple platforms in cases like this was a making-out strategy for expanding one's possibilities to find interesting assignment and smoothing out income stream (Table 3: quote 2). As stated above, many freelancers on Gamma also sought clients from alternative sources. Unlike experts on Delta, their main motivation for doing so was to bypass the platform's high service fee.

#### 4.3.2. Taking on new roles

Some freelancers are not registered on Gamma only as “talents” but also as clients. This is perfectly permissible, like in cases where freelancers outsource parts of their own assignments to other freelancers. However, in some cases, freelancers' underlying motives for doing this may work against corporate intentions and be seen as acts of making out by distorting competition among freelancers. By taking on the role of “artificial” client, freelancers can strive to improve their success in the competition for assignments by getting a more in-depth view of the platform's operations from the client's point of view as well as their freelancer competitors' competences and application strategies (Table 3: quote 3).

#### 4.3.3. Tricks

In some cases, workers use “tricks,” i.e., practices that are not in line with the interests or rules of the platform to promote their own interests. One courier used Alpha's backpack even when gigging for Beta. The courier preferred Alpha's backpack because it was more comfortable and practical, justifying his behavior with a lack of commitment to the platforms (Table 3: quote 4). There was also another case where a courier wore “wrong” gear for the same reason. This lack of commitment, which lowers couriers' threshold for gaming, also comes out apply in the following quote: “*Every time I'm in my car I have both bags.”*

Many of the tricks are efforts to game algorithmic management and the way it affects workers' assignments and rankings. Couriers on Alpha are automatically put on forced break if they reject three invitations in a row. However, they have learned to work around this by taking a break at an appropriate time and logging in again (Table 3: quote 5). At Gamma, the score has a significant impact on freelancers' conditions for receiving assignments from clients, but, at the same time, their opportunities to fully influence it are limited. One common way to influence one's score is to carefully select what kind of clients and projects one takes on. This is not an act of making out as such. It is possible for a freelancer to stop the project if he/she believes that he/she would receive a bad client review. This kind of strategy mainly comes into question in hourly projects, where a freelancer has received payment for the work already done, and it cannot be used without justification and too often. A freelancer can also “just in case” give good reviews of clients to increase their chances of getting new projects from them, or knowingly “order” good reviews from acquaintances (Table 3: quote 6).

#### 4.3.4. Disintermediation

Taking the client outside the platform can be considered the most radical form of making out that emerged in our material. Many of the platforms' controlling practices specifically aim to prevent this (see also Zhu and Iansiti, 2019). The business model of the platforms is based both on the service fees they receive from transactions *via* the platforms and on the information superiority and the consequent strengthening of their market position that grows with each new transaction in relation to workers, clients, and potential competitors. Disintermediation is not permitted on Gamma and Delta. On Gamma, some freelancers still do it in all silence while they continue working *via* Gamma with other clients (Table 3: quote 7). Instead, among interviewed experts on Delta, examples of such radical acts of making out did not come up. In cases of disintermediation, the clients had paid a recruitment fee to Delta in accordance with official contract protocol.

## 5. Discussion and conclusions

Here, we first reflect on our findings regarding the different types. Thereafter, we present the key theoretical and practical contributions of the paper. Finally, we highlight the limitations of our study and the related future research needs.

### 5.1. Reflection on the findings

According to our empirical analysis, in type 1 workers' acts of agency are largely oriented toward questioning. In the light of the assumptions presented in Section 2, the high level of standardization of the work process combined with workers' lack of knowledge of how the algorithm allocates tasks and ranks them limits in many ways their opportunities for moving from mere questioning to actual making out. Perrig (2021) talks of gamification-from-above in the context of platform work, referring to algorithmic control that gives workers enough information to participate in the game (to direct their behavior) but not enough to be able to game the system. By collecting vast amounts of data from all stages of the work process, food-delivery platforms can continuously fine-tune their algorithms—making them increasingly opaque to workers—and better direct workers' actions. Through this, the platforms can also better predict the amount of labor needed in different places at any given time and learn how to better match demand and supply with the help of behavioral and economic nudges. In this way, they can also raise the threshold for potential competitors to enter the market.

Couriers play a similar game of making out as manufacturing workers described by Roy (1954) and Burawoy (1979), but with changed rules. Manufacturing workers' gaming may have been individual, but it was supported by a well-established social organization of work. We found few examples of couriers' collective agency in our interview data. This finding also applies to forms of agency that Stewart et al. (2020) call—in the absence of workplace collectivism—work*space* collectivism. In this respect, our results resemble those of Veen et al. (2020) from Australia but differ from many other studies in Europe that include examples of self-organization and collective expressions of voice (e.g., Kellogg et al., 2020; Bronowicka and Ivanova, 2021; Heiland and Schaupp, 2021; Cini, 2023). This may be because many of the couriers in our data—but also in Finland in general—come from diverse immigrant backgrounds. Alpha used to have designated teams for couriers, coordinated by salaried team leaders. However, the teams were disbanded without warning and an actual stated reason, making horizontal communication between couriers more difficult. Official communication on both food-delivery platforms now takes place only between the courier and the platform.

The importance of an “appropriate” level of opacity of algorithmic control is emphasized even more in work that is not as easy to standardize (Rahman, 2021), exemplified in our analysis by type 2. Freelancers' practicing of agency in our data was manifested more often as acts of making do/out than mere questioning or ideating compared to couriers (Table 2) and revolving around reputational insecurities, which might overshadow their ability to access new assignments (cf. also Schörpf et al., 2017; Rahman, 2021; Wood and Lehdonvirta, 2022). Third-party evaluations are the basis of their online reputation, embodied in their score. However, besides public client feedback, the score is also affected by their private feedback requested by the platform; a fact that freelancers or even the clients are not necessarily aware of. The obscurity of algorithmic evaluation is further increased by the fact that freelancers do not fully know what factors are causing changes in their score. Freelancer platforms also can, like food-delivery platforms, use their constantly accumulating data reserves to increase or decrease the competition between freelancers in many ways, such as by applying filters or influencing the transaction fees.

Along with the level of work standardization, couriers and freelancers are distinguished by their future perspective. The job of a courier is a dead-end job in terms of learning or advancement. Couriers may socio-demographically greatly differ from each other, but as couriers, they are completely replaceable. The work of freelancers typically offers more opportunities for learning, and in addition, unlike couriers, they form a heterogeneous group also as platform workers. Some of them largely focus on their core competencies as freelancers, while others have a more entrepreneurial orientation (Nemkova et al., 2019). Entrepreneurial orientation can be most clearly seen in our data as a conscious strategy of some freelancers to increase their reputation, acquire regular long-term customer relationships, and, with the help of these, gradually leave the platform completely. Such a way of activity, i.e., disintermediation, can be considered perhaps the most apt example of transformative digital agency (Vänninen et al., 2015) in our data.

The experts operating through Delta (type 3) form a special group of platform workers—seldom, if ever, examined in studies on platform workers—which still differs from the previous ones. Delta's control structure is more transparent, and it is based on a high-trust relationship between the platform, clients, and experts. The platform has created a clearly different relationship with experts than Gamma with freelancers because the reputation of people who have held management positions is not built on the platform's client feedback, but on their entire work history from outside the platform. The experts' financial or other dependency on the platform is also less than the others' due to their typically greater wealth and larger social networks based on their previous work history. The permissive nature of the platform's control structure leaves much room for the experts' agency. However, their need to channel their agency into gaming behavior is limited by their closer, trust-based relationship with the platform. Unlike the previous two types, the platform does not appear to them so much as a faceless machine, but, rather, as an element in their overall social network.

### 5.2. Theoretical and practical contributions

Our analysis shows how workers operating *via* three different types of platforms have practiced their digital agency. The platforms differ in their control contexts, affecting the room they leave for workers' agency and its forms of expression. It is obvious that platform workers are not a homogeneous group (Vallas and Schor, 2020), and platform work is not just another layer of a periphery segment in the labor market (Kristiansen et al., 2022). Platforms exercising algorithmic control are new types of arenas for work, which, in line with earlier studies on the digital divides and inequalities (Helsper, 2012; Van Deursen et al., 2017), seem to reproduce the inequalities—for example, in work autonomy, skills development, career advancement, and labor market vulnerabilities—found in the offline world of work in the digital world. In our data, such inequalities manifest as different forms of control among the three platform archetypes, providing different opportunities for practicing agency for different groups of workers.

Showing the interplay between platform forms of control, the nature of work tasks, workers' opportunities for practicing agency, and their acts of making out, based on a comparative setting, can be considered the major contribution of this paper to existing research literature. As another contribution, the study brings out how platform work not only revolutionizes ways of working, but, as a Janus-faced phenomenon, also reproduces prevailing social relations in a new context. The differences found between food couriers, freelancers, and interim managers are largely mirror images of the differences that have already existed between groups of low-skilled workers, high-skilled professionals, and managers in the offline world of work with two major exceptions.

The first exception is related to the new technological affordances of algorithms. The ability of the platforms to accumulate data enables them to develop their algorithmic management in a way that increases their information superiority over *all* kinds of platform workers. This kind of redistribution of power, based on the platforms' growing information superiority, has potentially deteriorating effects also on the position of high-skilled groups in the future digital labor market.

The second exception concerns the position of groups of low-skilled workers on the platforms. The power of many blue-collar workers in the labor market has traditionally been based more on their workplace communities and trade unions than in the case of professionals or other white-collar workers. However, as already implicated, the way food-delivery work is organized does not offer the couriers the same kind of “natural” social organization and the potential for collective agency as for blue-collar workers in more traditional settings. The loss of traditional collective sources of power, without new countervailing forms of organizing, has the potential to further increase their vulnerability in the labor market in relation to other groups of platform workers.

From the perspective of labor policy, both findings raise questions regarding the power advantages of platforms over workers in the labor market and the cementing, or even amplifying, effects of platform work on inequalities between different groups of workers.

### 5.3. Limitations and future research ideas

Economic activities are not immune to institutional context. Our analysis focused on Finland where labor markets and industrial relations are highly regulated like in the other Nordic countries. Earlier studies have shown that, in such environments, platform companies experience pressure from social partners and legislators, having implications for their freedom of action (Jesnes, 2019; Oppegaard et al., 2020). However, we believe that the national context has not had a major impact on the results in this case. The procedures of both food-delivery platforms seem very similar to those found in earlier studies dealing with similar work in other industrial countries. Gamma also operates around the world with uniform procedures. Delta's operations are limited to Finland. However, as stated above, it is difficult to find examples in the research literature focusing on this kind of platform work in other countries. This notwithstanding, institutional factors may in different ways limit platform companies' freedom of operation in different national contexts, an area where more research is needed.

Another possible factor influencing the results could have been how the interviewees were selected. It is not possible to estimate to what extent the group of interviewees corresponds statistically to the total group of people involved in platform work in Finland, because we are currently lacking such a data basis in Finland. The group of interviewees is quite diverse in each type in socio-demographic background, labor market status, and platform activity. This suggests that the material would not be clearly biased according to any single background factor.

We did not examine in more detail how financially dependent workers are on the platform. Rahman (2021) has presented that platform dependence together with evaluation setbacks is connected to how experimental vs. constrained the workers' activity on the platform are. As shown in Section 3, the dependence of the interviewed workers on the earnings *via* the platforms probably differs clearly from one another. This may have had an impact on how *individual* workers have acted on the platforms. However, the subject of the study was not the workers' different behaviors in itself, but the impact of the different platform control structures on the conditions for workers' agency.

In our study, the notion of digital agency was repurposed for a sociological inquiry. The analysis focused on acts of making out, which is a concept that originates from sociological studies revolving around workers' resistance toward management or the work system (e.g., Roy, 1954; Burawoy, 1979). However, workers' agency can emerge and evolve also from other kinds of motives. While seemingly useful in our analysis in the context of platform work, our interpretation of platform workers' digital agency may have left unnoticed other more developmental aspects of platform work that uncover potentials for workers' learning in complex digital ecosystems. The purpose for which platform workers practice or can practice their digital agency—beyond making out—is an important topic for future research.

Finally, our study is limited in the sense that we do not know enough of the underlying intentions of platform companies, which makes it increasingly difficult to draw a line between acts of making do and making out in platform work. Our understanding is obscured particularly by two factors. First, the business principles of platform companies may differ considerably from those of more traditional companies. This is aptly brought out, for example, in the characterization of Van Doorn and Badger (2021) on how platform companies capture both monetary value associated with the services they produce and more speculative and volatile types of value based on the data they generate in connection with the provision of services. The companies' algorithms—which can be considered the most authentic reflection of these principles—are beyond our reach. The opacity of algorithms to workers or any outside observers may be intentionally built in them, as already alluded to above, but it may also be due to their machine learning-based capacity or other technological features in themselves (Kellogg et al., 2020). Second, platform companies' intentions can also be influenced by factors other than purely business-related considerations, such as efforts to increase their social legitimacy or to prevent increasing regulation of their operations in advance. To increase our understanding, multidisciplinary research approaches that can better combine sociological, educational, business management, and technological (data science) expertise are welcome in future studies on platform work.

## Data availability statement

The datasets presented in this article are not readily available because The interview text data that form the key data source for this study is not publicly available due to the privacy agreement that signed between the interviewer and the interviewee before the interview and its recording and transcription. According to the agreement, the recordings and their transcriptions collected in the studies are treated as strictly confidential and in all stages of the studies they are only for the knowledge and use of the research teams of the studies. Requests to access the datasets should be directed to tuomo.alasoini@ttl.fi.

## Ethics statement

Ethical review and approval was not required for the study on human participants in accordance with the local legislation and institutional requirements. The patients/participants provided their written informed consent to participate in this study.

## Author contributions

TA, JI, LS, and MK contributed equally to the design of the research question and research tasks. JI made an analysis of platform webpages and conducted interviews of workers on the Delta platform and those for whom Beta was their main platform. LS conducted interviews of workers on the Gamma platform and those for whom Alpha was their main platform. JI, LS, and MK made the interview text data analysis and wrote individual sections of the results to the manuscript. TA wrote the first draft of the manuscript. All authors contributed to manuscript revision, read, and approved the submitted version.
